# Alcohol consumption and breast cancer oestrogen and progesterone receptor status

**DOI:** 10.1038/sj.bjc.6690210

**Published:** 1999-03

**Authors:** S M Enger, R K Ross, A Paganini-Hill, M P Longnecker, L Bernstein

**Affiliations:** Research and Evaluation Department, Kaiser Permanente Medical Care Program, Pasadena, Southern California, CA 91188, USA; Department of Preventive Medicine, University of Southern California and Norris Comprehensive Cancer Center, Los Angeles, CA 90033, USA; Epidemiology Branch, National Institute of Environmental Health Sciences, Research Triangle Park, NC 27709, USA

**Keywords:** alcohol, oestrogen receptors, progesterone receptors, breast neoplasms, epidemiology

## Abstract

We examined the role of alcohol on the risk of breast cancer by the joint oestrogen receptor (ER) and progesterone receptor (PR) status of the tumour using data from two case-control studies conducted in Los Angeles County, USA. Eligible premenopausal patients were 733 women aged ≤ 40 years and first diagnosed from 1 July 1983 to 1 January 1989. Eligible postmenopausal patients were 1169 women aged 55–64 years and first diagnosed from 1 March 1987 to 31 December 89. Patients were identified by the University of Southern California Cancer Surveillance Program. Neighbourhood controls were individually matched to patients by parity (premenopausal patients) and birth date (± 3 years). ER and PR status were obtained from medical records for 424 premenopausal and 760 postmenopausal patients. The analyses included 714 premenopausal and 1091 postmenopausal control subjects. Alcohol use was generally not associated with premenopausal risk of breast cancer, regardless of hormone-receptor status. Among the postmenopausal women, those who consumed, on average, ≥ 27 g of alcohol/d experienced an odds ratio (OR) of 1.76 [95% confidence interval (CI) 1.14–2.71] for ER-positive/PR-positive breast cancer relative to women who reported no alcohol consumption. Alcohol use was less clearly associated with risk of other receptor types among postmenopausal women. These data suggest that alcohol may preferentially increase risk of ER-positive/PR-positive breast cancer in postmenopausal women. © 1999 Cancer Research Campaign


*British Journal of Cancer* (1999) **79**(7/8), 1308–1314
© 1999 Cancer Research Campaign
Article no. bjoc.1998.0210

### Alcohol consumption and breast cancer oestrogen and progesterone receptor status

**SM Enger[1,2], RK Ross[2], A Paganini-Hill[2], MP Longnecker[3] and L Bernstein[2]**

[1]Research and Evaluation Department, Kaiser Permanente Medical Care Program, Southern California, Pasadena, CA 91188, [2]Department of Preventive Medicine, University of Southern California and Norris Comprehensive Cancer Center, Los Angeles, CA 90033 and [3]National Institute of Environmental Health Sciences, Epidemiology Branch, Research Triangle Park, NC 27709, USA

**Summary** We examined the role of alcohol on the risk of breast cancer by the joint oestrogen receptor (ER) and progesterone receptor (PR) status of the tumour using data from two case-control studies conducted in Los Angeles County, USA. Eligible premenopausal patients were 733 women aged ≤ 40 years and first diagnosed from 1 July 1983 to 1 January 1989. Eligible postmenopausal patients were 1169 women aged 55–64 years and first diagnosed from 1 March 1987 to 31 December 89. Patients were identified by the University of Southern California Cancer Surveillance Program. Neighbourhood controls were individually matched to patients by parity (premenopausal patients) and birth date (± 3 years). ER and PR status were obtained from medical records for 424 premenopausal and 760 postmenopausal patients. The analyses included 714 premenopausal and 1091 postmenopausal control subjects. Alcohol use was generally not associated with premenopausal risk of breast cancer, regardless of hormone-receptor status. Among the postmenopausal women, those who consumed, on average, ≥ 27 g of alcohol/d experienced an odds ratio (OR) of 1.76 [95% confidence interval (CI) 1.14–2.71] for ER-positive/PR-positive breast cancer relative to women who reported no alcohol consumption. Alcohol use was less clearly associated with risk of other receptor types among postmenopausal women. These data suggest that alcohol may preferentially increase risk of ER-positive/PR-positive breast cancer in postmenopausal women.

**Keywords:** alcohol; oestrogen receptors; progesterone receptors; breast neoplasms; epidemiology

Over the past 2 decades, numerous studies have provided substantial evidence for an association of alcohol consumption and risk of breast cancer (Longnecker, 1994). Research has recently focussed on the role of steroid hormone receptors in the association of alcohol with breast cancer, given evidence suggesting that alcohol consumption increases endogenous oestrogen levels (Singletary, 1996) and given the role of oestrogen receptors (ERs) in facilitating the breast cell-stimulating activity of oestrogen (Stanford et al, 1986; Habel and Stanford, 1993). Results from a large breast cancer case-control study suggested that the increased risk of breast cancer associated with alcohol consumption may be restricted to ER- positive tumours (Nasca et al, 1994), a finding that was not consistent with results of other studies of the issue (McTiernan et al, 1986; Cooper et al, 1989; Holm et al, 1989; Potter et al, 1995; Yoo et al, 1997). This issue remains unresolved because of limited statistical power in some of the earlier studies and a lack of information on the joint ER and progesterone receptor (PR) status of the patients in all but two of the studies (Potter et al, 1995; Yoo et al, 1997). A number of studies have demonstrated that the joint ER and PR status may have greater prognostic value than either ER or PR separately (McGuire, 1986; Ruder et al, 1989; Gamulin and Romic-Stojkovic, 1991; Raabe et al, 1998).

*Received 5 August 1998*
*Revised 6 October 1998*
*Accepted 7 October 1998*

*Correspondence to:* SM Enger, Department of Research and Evaluation, Kaiser Permanente Medical Care Program, 100 S. Los Robles Avenue, Second Floor, Pasadena, CA 91188, USA

Clarification of the association of alcohol with breast tumour hormone receptor status may improve our understanding of the role of alcohol in breast cancer aetiology. Using data from two population-based case-control studies of breast cancer risk factors in Los Angeles County, we examined the association of alcohol and risk of breast cancer according to joint ER and PR status.

### MATERIALS AND METHODS

### Subjects

Subjects eligible to participate were English-speaking, white (including Hispanic), female residents of Los Angeles County, born in the USA, Canada, or Western Europe, with no history of breast cancer. Eligible case subjects were all patients aged 40 years or younger first diagnosed between 1 July 1983 and 1 January 1989, with histologically confirmed in situ or invasive breast cancer and all patients aged 55–64 years first diagnosed between 1 March 1987 and 31 December 1989 with histologically confirmed in situ or invasive breast cancer. Case subjects were identified by the University of Southern California Cancer Surveillance Program (CSP), the population-based cancer registry for Los Angeles County. These study populations have been described in detail elsewhere (Bernstein et al, 1994; Longnecker et al, 1995).

Briefly, a total of 744 (77%) of 969 eligible patients aged 40 years or younger completed the interview. Of the 225 eligible case subjects who did not participate, the physician refused to allow contact with 54 (6% of eligible patients), 27 (3%) could not be interviewed because of mental or physical health problems or because they had died, 111 (11%) refused to be interviewed, 12

(1%) moved out of Los Angeles County and could not be interviewed in person, and 21 (2%) were lost to follow-up.

One control subject was individually matched by birth date (within 3 years), parity (nulliparous vs parous) and neighbourhood to each of the 744 young breast cancer patients who completed the interview. Control subjects were selected from housing units in a pre-defined walk pattern in the neighbourhood where the case lived at the time of her breast cancer diagnosis. The response rate among eligible controls was 79% based on the total number of controls we attempted to recruit in order to recruit 744 successfully.

A total of 1579 (67%) of 2373 eligible patients aged 55–64 years completed the interview. Physicians recommended against our contacting 128 (5% of eligible subjects) patients, 419 (18%) patients refused to be interviewed, 230 (10%) patients were too ill or had died and we were unable to locate 17 (< 1%) patients.

One control subject was individually matched to 1506 of the 1579 interviewed breast cancer patients on birth date (within 3 years) and neighbourhood of residence. Control recruitment was handled in the same manner as for the study of younger women. We were unable to identify and interview an eligible control for the remaining 73 case subjects. The response rate among eligible control subjects was 80%.

### Demographics

Detailed information regarding demographic characteristics and reproductive histories as well as other known or suspected breast cancer risk factors was obtained by face-to-face interview with each subject. For each case and control pair (and for unmatched case patients in the study of older women), a reference date was created that was the date 12 months before the index patient's breast cancer diagnosis. Information obtained by interview includes only those exposures that occurred before the reference date.

### Alcohol consumption

The participants were queried about the number of drinks of beer, wine and liquor that they consumed per week on average at ages 18, 25 and the reference age (women aged 40 years or younger) and at ages 25, 40 and the reference age (women aged 55–64). Results pertaining to alcohol consumption before the reference age added little information and are not shown. We calculated the average number of grams of alcohol consumed per day as the number of drinks per day for each type of alcoholic beverage multiplied by the estimated grams of alcohol in each beverage, which we assumed to be 12.8 for one serving of beer, 10.9 for one 4 oz glass of wine and 15.0 for one mixed drink (USDA, 1986).

### Exclusions

For the purposes of this study, we excluded 11 case patients and 16 control subjects from the study of younger women because the women were no longer menstruating, and we excluded 13 case patients and 14 control subjects who did not know their family history of breast cancer because they had been adopted. A total of 720 case patients and 714 control subjects remained in the study of pre-menopausal women. In addition, we excluded 419 case patients (of the 1579 matched and unmatched case patients) and 415 control subjects from the study of older women for the following reasons: premenopausal (still menstruating and not using hormone-replacement therapy: 58 case patients and 51 control subjects), unknown age at menopause (usually hysterectomy without bilateral oophorectomy: 352 case patients and 360 control subjects) or incomplete information on family history, education, alcohol consumption, pregnancies, breastfeeding or weight (nine case patients and four control subjects). A total of 1160 case patients and 1091 control subjects remained in the study of post-menopausal women.

**Table 1** Joint distribution of ER and PR status among breast cancer patients with known tumour hormone receptor status

| Joint ER/PR status | Premenopausal Frequency (*n* = 424) | % | Postmenopausal Frequency (*n* = 760) | % |
|---|---|---|---|---|
| ER+/PR+ | 205 | (49) | 450 | (59) |
| ER+/PR– | 52 | (12) | 159 | (21) |
| ER–/PR+ | 18 | (4) | 24 | (3) |
| ER–/PR– | 149 | (35) | 127 | (17) |

Positive = +; negative = –.

### ER and PR status

CSP abstracts which include copies of the patients' pathology reports were reviewed for ER and PR status (positive or negative) for each breast cancer patient in the two studies. Medical and pathology records were requested and reviewed at the hospital of diagnosis if the information was not included in the CSP records for the patient. In both studies, for over 50% of the women who had missing receptor status data, the charts were located but results of the receptor assays, if done, were not in the record. The chart was unavailable, generally due to destruction or hospital closure, for about one-third of the women with missing data. ER or PR status, but not both, was available for about 10% of the women with missing data.

Of the 720 premenopausal patients with complete interview information, we retrieved ER status for 441 (61%), PR status for 425 (59%) and joint ER/PR status for 424 (59%). A total of ten patients had ER-positive tumours and seven had ER-negative tumours but were missing PR status and one patient had a PR-positive tumour but was missing ER status. We have included 405 case patients in the statistical analyses whose tumours were known to be ER-positive/PR-positive, ER-positive/PR-negative or ER-negative/PR-negative [there were too few women with ER-negative/PR-positive tumours (*n* = 19) to permit useful analyses], 296 women with unknown tumour ER or PR status and 714 control subjects.

We retrieved ER status for 805 (69%), PR status for 760 (66%) and joint ER/PR status for 760 (66%) of the 1160 postmenopausal patients with complete interview information included in the study of women 55–64 years of age. A total of 34 women had ER-positive tumours and 11 had ER-negative tumours but were missing PR status, and no women had PR status but were missing ER status. We included 736 case patients whose tumours were known to be ER-positive/PR-positive, ER-positive/PR-negative, or ER-negative/PR-negative [there were too few women with ER-negative/PR-positive tumours (*n* = 24) to permit useful analysis], 400

© Cancer Research Campaign 1999

women with unknown tumour ER or PR status, and 1091 control subjects in the statistical analyses.

### Statistical analyses

We compared the risk factor distributions for patients with known ER and PR status to those of patients whose ER and PR status was unknown using the $\chi^2$ test. ORs and 95% CIs were calculated to estimate the breast cancer risk associated with alcohol consumption using unconditional logistic regression methods within joint ER and PR status subgroups using four separate models as follows: ER-positive/PR-positive vs controls, ER-positive/PR-negative vs controls, ER-negative/PR-negative vs controls, and ER unknown/PR unknown vs controls. Pair matching was not retained in the analyses in order to maximize the number of subjects to be included in the analyses. The results were not materially different when the matching was retained. We used the two-sided *P*-value associated with the coefficient fit to the median value of each category of the variable to test for trend in effect across categories of a risk factor. We used polytomous logistic regression analysis to test for heterogeneity in the effect of alcohol consumption as a continuous variable across response functions of each of the hormone receptor status subgroups, with the control subjects serving as the reference group. SAS statistical software was used to perform all statistical analyses (SAS Institute, Cary, NC, USA).

We included the matching variables [age at reference year (continuous variable) and socio-economic status (five categories based on census tract of residence)] as covariates in the multivariate models for both studies. For premenopausal women, we

**Table 2** Distribution of patient and tumour factors among patients with known and missing tumour hormone receptors status

| | Premenopausal | | | | | Postmenopausal | | | | |
|---|---|---|---|---|---|---|---|---|---|---|
| | ER and PR known | | ER or PR missing | | | ER and PR known | | ER or PR missing | | |
| **Factor** | ***n*** | **%** | ***n*** | **%** | ***P*** | ***n*** | **%** | ***n*** | **%** | ***P*** |
| Age at diagnosis (years) | | | | | | | | | | |
| 21–25 | 8 | 2.0 | 7 | 2.3 | | | | | | |
| 26–30 | 30 | 7.4 | 20 | 6.6 | | | | | | |
| 31–35 | 134 | 33.0 | 82 | 27.2 | | | | | | |
| 36–40 | 234 | 57.6 | 193 | 63.9 | 0.25 | | | | | |
| 55–59 | | | | | | 313 | 42.5 | 177 | 44.3 | |
| 60–64 | | | | | | 423 | 57.5 | 223 | 55.8 | 0.57 |
| Stage | | | | | | | | | | |
| In situ | 16 | 3.9 | 50 | 16.6 | | 17 | 2.3 | 91 | 22.7 | |
| Localized | 195 | 48.0 | 137 | 45.4 | | 440 | 59.8 | 226 | 56.5 | |
| Regional | 173 | 42.6 | 98 | 32.4 | | 262 | 35.6 | 74 | 18.5 | |
| Metastatic | 11 | 2.7 | 9 | 3.0 | | 16 | 2.2 | 5 | 1.3 | |
| Unstageable | 11 | 2.7 | 8 | 2.6 | 0.001 | 1 | 0.1 | 4 | 1.0 | 0.001 |
| Age at menarche (years) | | | | | | | | | | |
| < 12 | 105 | 25.9 | 91 | 30.1 | | 145 | 19.7 | 85 | 21.3 | |
| 12 | 119 | 29.3 | 85 | 28.1 | | 191 | 26.0 | 108 | 27.0 | |
| 13 | 116 | 28.6 | 87 | 28.8 | | 230 | 31.3 | 107 | 26.8 | |
| ≥ 14 | 66 | 16.3 | 39 | 12.9 | 0.45 | 170 | 23.1 | 100 | 25.0 | 0.39 |
| Age at first full-term pregnancy (years) | | | | | | | | | | |
| Never pregnant | 152 | 37.4 | 116 | 38.4 | | 120 | 16.3 | 61 | 15.3 | |
| < 20 | 55 | 13.5 | 43 | 14.2 | | 95 | 12.9 | 48 | 12.0 | |
| 20–24 | 95 | 23.4 | 58 | 19.2 | | 287 | 39.0 | 152 | 38.0 | |
| 25–29 | 69 | 17.0 | 56 | 18.5 | | 151 | 20.5 | 93 | 23.3 | |
| ≥ 30 | 35 | 8.6 | 29 | 9.6 | 0.74 | 83 | 11.3 | 46 | 11.5 | 0.87 |
| Number of full-term pregnancies | | | | | | | | | | |
| 0 | 152 | 37.4 | 116 | 38.4 | | 120 | 16.3 | 61 | 15.3 | |
| 1 | 74 | 18.2 | 61 | 20.2 | | 104 | 14.1 | 45 | 11.3 | |
| 2 | 123 | 30.3 | 79 | 26.2 | | 208 | 28.3 | 117 | 29.3 | |
| 3 | 38 | 9.4 | 30 | 9.9 | | 156 | 21.2 | 103 | 25.8 | |
| ≥ 4 | 19 | 4.7 | 16 | 5.3 | 0.71 | 148 | 20.1 | 74 | 18.5 | 0.31 |
| Age at menopause | | | | | | | | | | |
| < 45 | | | | | | 124 | 16.8 | 52 | 13.0 | |
| 45–49 | | | | | | 187 | 25.4 | 111 | 27.8 | |
| 50–54 | | | | | | 344 | 46.7 | 184 | 46.0 | |
| ≥ 55 | | | | | | 81 | 11.0 | 53 | 13.3 | 0.20 |
| Family history | | | | | | | | | | |
| No | 357 | 87.9 | 237 | 78.5 | | 612 | 83.2 | 315 | 78.8 | |
| Yes | 44 | 10.8 | 57 | 18.9 | | 124 | 16.8 | 85 | 21.3 | |
| Unknown | 5 | 1.2 | 8 | 2.6 | 0.002 | | | | | 0.07 |

© Cancer Research Campaign 1999

also included as categorical variables the following factors: age at menarche (< 12, 12, 13, ≥ 14 years), age at first full-term pregnancy (never, < 20, 20–24, 25–29, ≥ 30 years), number of full-term pregnancies (0, 1, 2, 3, ≥ 4), lifetime months of breastfeeding (0, 1–6, 7–15, ≥ 16), years of use of oral contraceptives (0, 1–4, 5–9, ≥ 10), average hours per week of physical activity after menarche (0, 0.1–0.7, 0.8–1.6, 1.7–3.7, ≥ 3.8) and first-degree family history of breast cancer (yes/no).

For postmenopausal women, we included as categorical variables in all multivariate models age at menarche (< 12, 12, 13, ≥ 14 years), age at first full-term pregnancy (never, < 20, 20–24, 25–29, ≥ 30 years), number of full-term pregnancies (0, 1, 2, 3, ≥ 4), lifetime months of breastfeeding (0, 1–3, 4–6, 7–15, ≥ 16), age at menopause (< 45, 45–49, 50–54, ≥ 55 years), use of oestrogen-only hormone-replacement therapy (0, 1–12, 13–72, 73–120, ≥ 121 months), use of combined oestrogen and progestin hormone-replacement therapy (0, 1–12, 13–72, 73–120, ≥ 121 months), body-mass index (BMI) at the reference date (< 21.8, 21.8–23.9, 24.0–27.3, ≥ 27.4 kg/m$^2$), physical activity (a combination variable based on average MET-hours (the ratio of the metabolic rate associated with a given activity to the resting metabolic rate) per week (MH) of activity before/after age 40: 0/0, low/low (at least one is > 0), low/high, high/low, high/high, where low is < 17.6, and high is ≥ 17.6 MH), education (less than high school, high school, partial college, college or graduate/professional training) and first-degree family history of breast cancer (yes/no).

### RESULTS

As expected, a greater proportion of post-menopausal than pre-menopausal patients had tumours that expressed both ER and PR, while twice the proportion of pre-menopausal compared with post-menopausal patients had tumours that expressed neither ER nor PR (Table 1). Tumours that expressed PR but not ER were rare in both groups.

In general, the distributions of various patient factors were not different between the group of patients for whom receptor status was ascertained and the group with missing receptor status information (Table 2). However, we observed that a greater proportion of patients with unknown than with known hormone-receptor status had in situ tumours, probably due to insufficient tissue for the receptor assays. Among premenopausal patients with unknown receptor status, the proportion with in situ disease was fourfold greater than in those with disease of known receptor status. Among postmenopausal breast cancer patients, the proportion of in situ cases with unknown receptor status was nearly tenfold greater. We also observed that a greater proportion of premenopausal and postmenopausal patients with missing receptor status had a first-degree family history of breast cancer than did patients whose receptor status was ascertained.

Among premenopausal women with known receptor status, alcohol consumption was generally unrelated to risk of breast cancer regardless of receptor type (Table 3). Because the premenopausal women consumed fairly low levels of alcohol, the highest category of consumption in these analyses was 14 g of alcohol per day or more (approximately one drink). An association of alcohol consumption with risk of breast cancer was observed, however, among cases with unknown receptor status.

Among post-menopausal women, alcohol consumption was associated more with increased risk of ER-positive/PR-positive breast cancer than with tumours of other receptor type (Table 3). Women who consumed at least 27 g/day on average (approximately two drinks) in the recent past experienced more than a 75% increase in risk of ER-positive/PR-positive breast cancer. We obtained similar findings for alcohol consumption at age 40 and for maximum alcohol consumption (the maximum of ages 25, 40 and the reference age) (results not shown). The results were not materially different when analysed for invasive tumours only (not shown). The alcohol coefficients compared across receptor status subgroups showed no statistically significant differences in polytomous logistic regression analyses with alcohol consumption modelled as a continuous variable.

**Table 3** Association of alcohol consumption with breast cancer risk, according to joint oestrogen receptor (ER) and progesterone receptor (PR) status[a]

| | | ER+/PR+ | | | ER+/PR– | | | ER–/PR– | | | ER unknown/PR unknown | | |
|---|---|---|---|---|---|---|---|---|---|---|---|---|---|
| **Alcohol (g/day)** | **Controls** | **Cases** | **OR[b]** | **(95% CI)** | **Cases** | **OR[b]** | **(95% CI)** | **Cases** | **OR[b]** | **(95% CI)** | **Cases** | **OR[b]** | **(95% CI)** |
| Premenopausal | | | | | | | | | | | | | |
| 0 | 385 | 110 | 1.00 | | 37 | 1.00 | | 85 | 1.00 | | 157 | 1.00 | |
| 1–5 | 135 | 30 | 0.73 | (0.46–1.15) | 6 | 0.45 | (0.18–1.10) | 20 | 0.68 | (0.40–1.16) | 51 | 0.97 | (0.66–1.43) |
| 6–13 | 118 | 37 | 1.07 | (0.69–1.65) | 2 | 0.16 | (0.04–0.69) | 23 | 0.90 | (0.53–1.51) | 48 | 1.01 | (0.68–1.52) |
| 14+ | 88 | 28 | 1.10 | (0.67–1.80) | 7 | 0.71 | (0.30–1.68) | 21 | 1.04 | (0.60–1.81) | 46 | 1.27 | (0.83–1.94) |
| Trend P | | | 0.56 | | | 0.21 | | | 0.84 | | | 0.29 | |
| OR per 13 g | | | 1.10 | (0.91–1.32) | | 0.88 | (0.59–1.30) | | 1.08 | (0.89–1.31) | | 1.18 | (1.03–1.37) |
| Postmenopausal | | | | | | | | | | | | | |
| 0 | 590 | 239 | 1.00 | | 90 | 1.00 | | 71 | 1.00 | | 236 | 1.00 | |
| 1–13 | 329 | 122 | 0.97 | (0.74–1.27) | 38 | 0.75 | (0.49–1.14) | 33 | 0.81 | (0.52–1.26) | 95 | 0.75 | (0.56–1.00) |
| 14–26 | 109 | 46 | 1.18 | (0.80–1.75) | 21 | 1.36 | (0.80–2.33) | 12 | 0.91 | (0.47–1.75) | 34 | 0.84 | (0.54–1.29) |
| 27+ | 63 | 43 | 1.76 | (1.14–2.71) | 10 | 1.10 | (0.53–2.26) | 11 | 1.37 | (0.68–2.76) | 35 | 1.43 | (0.90–2.27) |
| Trend P | | | 0.03 | | | 0.65 | | | 0.77 | | | 0.79 | |
| OR per 13 g | | | 1.13 | (1.01–1.25) | | 1.05 | (0.90–1.24) | | 0.96 | (0.79–1.18) | | 1.06 | (0.95–1.18) |

[a]There were too few women with ER–/PR+ tumours to permit useful analysis (see methods). [b]Odds ratios adjusted for age at reference year, socioeconomic status, education, age at menarche, age at first full-term pregnancy, parity, lifetime months of breastfeeding, physical activity and family history. In the analysis that included pre-menopausal women, years of use of oral contraceptives was also included in the multivariate models. In the analysis that included postmenopausal women, age at menopause, oestrogen-only replacement therapy, combined oestrogen and progestin-replacement therapy and BMI were also included in the multivariate models (see Methods).

*© Cancer Research Campaign 1999*

**Table 4** Interactions of alcohol consumption with body-mass index (BMI) and with oestrogen-replacement therapy (ERT) use in relation to ER+/PR+ breast cancer risk (postmenopausal women)

| | Alcohol intake (g/d) | ER+/PR+ Cases | Controls | OR | (95% CI) |
|---|---|---|---|---|---|
| BMI[a] | | | | | |
| Low | | | | | |
| | 0 | 71 | 258 | 1.00 | |
| | 1–13 | 58 | 178 | 1.15 | (0.77–1.72) |
| | 14+ | 42 | 103 | 1.49 | (0.95–2.34) |
| High | | | | | |
| | 0 | 168 | 332 | 1.96 | (1.41–2.74) |
| | 1–13 | 64 | 151 | 1.61 | (1.08–2.41) |
| | 14+ | 47 | 69 | 2.53 | (1.59–4.04) |
| *P* for interaction[b] | | | | 0.52 | |
| ERT use[c] | | | | | |
| Never | | | | | |
| | 0 | 121 | 319 | 1.00 | |
| | 1–13 | 59 | 168 | 1.05 | (0.72–1.54) |
| | 14+ | 45 | 83 | 1.52 | (0.98–2.34) |
| Ever | | | | | |
| | 0 | 118 | 271 | 1.30 | (0.95–1.78) |
| | 1–13 | 63 | 161 | 1.17 | (0.80–1.78) |
| | 14+ | 44 | 89 | 1.66 | (1.07–2.58) |
| *P* for interaction[b] | | | | 0.47 | |

[a]Cutpoint for low vs high BMI: 23.7. [b]Interaction *P*-values determined from likelihood ratio test with 1° of freedom. [c]Oestrogen-replacement therapy use includes use of oestrogen-only-replacement therapy and combined oestrogen and progestin-replacement therapy.

We evaluated potential interactions of alcohol consumption with BMI and with ostrogen-replacement therapy (ERT) use for ER-positive/PR-positive tumours among postmenopausal women, because these breast cancer risk factors also represent oestrogen exposures (Table 4). We found that risk of ER-positive/PR-positive breast cancer was increased among the heaviest women (women in the ‘high’ BMI category) regardless of alcohol consumption level. We observed a 2.5-fold increase in ER-positive/PR-positive risk of breast cancer among heavier women who consumed at least 14 g of alcohol per day on average in the recent past compared to thinner women who did not consume alcohol. Among thinner women, consumption of 14 g of alcohol per day was associated with about a 50% increase in risk of ER-positive/PR-positive tumours compared to non-drinkers. Because the increase in risk of breast cancer across levels of alcohol intake was similar for the low and high BMI groups, evidence of effect modification by BMI for ER-positive/PR-positive breast cancer was not compelling. In a similar analysis, we observed no evidence of effect modification by ERT use of the association of alcohol consumption with ER-positive/PR-positive breast cancer.

### DISCUSSION

We observed an increased risk of ER-positive/PR-positive breast cancer among postmenopausal women who reported consumption of high levels of alcohol (> 27 g/day). Alcohol consumption was not associated with other hormone receptor subtypes postmenopausally and was generally not related to risk of any specific subtype among premenopausal patients. These results are generally consistent with those from a large case-control study conducted in New York, USA (Nasca et al, 1994), which included more than 1100 premenopausal and postmenopausal patients with breast cancer. In that study, Nasca and colleagues reported an increased risk of ER-positive, but not ER-negative, breast cancer at the highest levels of alcohol consumption. However, premenopausal and postmenopausal patients were combined and results for PR status were not presented. Five other studies have examined alcohol consumption in relation to risk of breast cancer by tumour hormone receptor status with highly mixed results: two reported no association of alcohol with tumour ER status (Cooper et al, 1989) or with joint ER/PR status (Yoo et al, 1997), one reported positive associations of alcohol with ER-positive and ER-negative tumours (McTiernan et al, 1986), one reported a modest increase in risk of ER-negative breast cancer with alcohol consumption (Holm et al, 1989), and one reported a positive association of alcohol consumption with risk of ER-negative/PR-negative breast cancer (Potter et al, 1995). All of the previous studies except one (Potter et al, 1995) presented the alcohol–receptor status results for both premenopausal and postmenopausal women combined. Previously reported findings that suggested an interaction between alcohol and BMI, and between alcohol and ERT use, within receptor status subgroups (Gapstur et al, 1995) were not replicated in our study.

Substantial epidemiological evidence supports an association of even modest alcohol consumption (one drink per day) with increased risk of breast cancer (Longnecker, 1994). In addition, results from experimental and cross-sectional data suggest that acute and chronic alcohol consumption increase endogenous oestrogen levels of both premenopausal and postmenopausal women (Mendelson et al, 1981, 1987, 1988, 1989; Teoh et al,

 © Cancer Research Campaign 1999

1988; Katsouyanni et al, 1991; Gavaler et al, 1993; Reichman et al, 1993; Dorgan et al, 1994; Hankinson et al, 1995). Overall, this evidence combined with results of a prospective study of endogenous oestrogens and postmenopausal breast cancer (Toniolo et al, 1995) indicate that the effect of alcohol on breast cancer risk may be mediated by oestrogen. However, analysis of data from the same prospective study revealed no clear associations between endogenous oestrogen levels and breast tumour hormone receptor status (Zeleniuch-Jacquotte et al, 1995), raising questions about the mechanisms underlying the association of alcohol consumption and ER-positive/PR-positive breast tumours.

Studies such as the one presented here may help clarify whether hormone receptor-positive and hormone receptor-negative breast tumours represent different stages in the progression of the disease or two distinct diseases with distinct aetiologies. The scientific literature includes evidence that can be interpreted to support either of these theories (Mobbs et al, 1987; Tani et al, 1988; Habel and Stanford, 1993; Zeleniuch-Jacquotte et al, 1995). Our finding that alcohol is more strongly associated with ER-positive/PR-positive risk of breast cancer among postmenopausal women supports the theory that hormone receptor status defines distinct diseases rather than different stages of the same disease.

This was the largest study to date to evaluate the association between alcohol consumption and risk of breast cancer by ER or PR type. Although generalization of findings from this study may be limited by the somewhat low ER and PR status recovery rates, it seems unlikely that the reasons for missing receptor status would have been related to receptor subtype or alcohol consumption patterns of the patients, especially since the majority of missing data was due to hospital policy (i.e. the destruction of charts inactive for over 7 years). Also, the distributions of most other breast cancer risk factors were generally similar for patients with known and unknown ER and PR status, and were therefore unlikely to introduce serious bias in the estimates derived from multivariate analyses. Another concern is that the hormone receptor status assays were performed in several different laboratories. However, results of two large European collaborative studies of steroid receptor distribution demonstrated that receptor assays were remarkably consistent across laboratories (Romain et al, 1995, 1996). In addition, the ER/PR distributions for both premenopausal and postmenopausal women in the present study are consistent with distributions reported from other studies (Bland et al, 1981; Thorpe, 1988; Potter et al, 1995).

In summary, these data suggest that alcohol may preferentially increase risk of ER-positive/PR-positive breast cancer in postmenopausal women, and that the association is not modified by BMI or ERT use. Although these findings support the hypothesis of an oestrogen-mediated effect of alcohol consumption on risk of breast cancer, further research is needed to determine whether the effects of alcohol consumption are mediated through interaction with steroid hormone receptors or through some other, possibly non-oestrogenic, pathway.

### ACKNOWLEDGEMENTS

The authors would like to thank Donna Morrell, CTR, and the technicians at the Cancer Surveillance Program of Los Angeles County for their invaluable assistance in collecting the hormone receptor status data. The authors also gratefully acknowledge the assistance of Ms Anjali Mahoney in the computer-entry of the hormone-receptor status data. Initial data collection for the two case-control studies was supported by Public Health Service grants CA17054 and CA44546 and contract N01-CN25404 from the National Cancer Institute, NIH, Department of Health and Human Services and by the California Public Health Foundation Subcontract 050-F-8709, which is supported by the California Department of Health Services as part of its state-wide cancer-reporting programme mandated by Health and Safety Code Sections 210 and 211.3. The ideas and opinions expressed herein are those of the authors, and no endorsement by the state of California, Department of Health Services, or the California Public Health Foundation is intended or should be inferred. Dr Enger was supported by funds from the California Breast Cancer Research Program of the University of California, Grant Numbers 1FB-0341 and 3FB-0097.

### REFERENCES

Bernstein L, Henderson BE, Hanisch R, Sullivan-Halley J and Ross RK (1994) Physical exercise and reduced risk of breast cancer in young women. *J Natl Cancer Inst* **86**: 1403–1408

Bland KL, Fuchs A and Wittliff JL (1981) Menopausal status as a factor in the distribution of estrogen and progestin receptors in breast cancer. *Surg Forum* **32**: 410–412

Cooper JA, Rohan TE, Cant EL, Horsfall DJ and Tilley WD (1989) Risk factors for breast cancer by oestrogen receptor status: a population-based case-control study. *Br J Cancer* **59**: 119–125

Dorgan JF, Reichman ME, Judd JT, Brown C, Longcope C, Schatzkin A, Campbell WS, Franz C, Kahle L and Taylor PR (1994) The relation of reported alcohol ingestion to plasma levels of estrogens and androgens in pre-menopausal women (Maryland, United States). *Cancer Causes Control* **2**: 53–60

Gamulin S and Romic-Stojkovic R (1991) Oestrogen and progesterone receptors in primary breast cancer: a population study. *Eur J Cancer* **27**: 491–495

Gapstur SM, Potter JD, Drinkard C and Folsom AR (1995) Synergistic effect between alcohol and estrogen replacement therapy on risk of breast cancer differs by estrogen/progesterone receptor status in the Iowa Women's Health Study. *Cancer Epidemiol Biomarkers Prev* **4**: 313–318

Gavaler JS, Deal SR, VanThiel DH, Arria A and Allan M (1993) Alcohol and estrogen levels in postmenopausal women: the spectrum of effect. *Alcohol Clin Exp Res* **17**: 786–790

Habel LA and Stanford JL (1993) Hormone receptors and breast cancer. *Epidemiol Rev* **15**: 209–219

Hankinson SE, Willett WC, Manson JE, Hunter DJ, Colditz GA, Stampfer MJ, Longcope C and Speizer FE (1995) Alcohol, height, and adiposity in relation to estrogen and prolactin levels in postmenopausal women. *J Natl Cancer Inst* **8717**: 1297–1302

Holm LE, Callmer E, Hjalmar ML, Lidbrink E, Nilsson B and Skoog L (1989) Dietary habits and prognostic factors in breast cancer. *J Natl Cancer Inst* **81**: 1218–1223

Katsouyanni K, Boyle P and Trichopoulos D (1991) Diet and urine estrogens among postmenopausal women. *Oncology* **48**: 490–494

Longnecker MP (1994) Alcoholic beverage consumption in relation to risk of breast cancer: meta-analysis and review. *Cancer Causes Control* **5**: 73–82

Longnecker MP, Paganini-Hill A and Ross RK (1995) Lifetime alcohol consumption and breast cancer risk among postmenopausal women in Los Angeles. *Cancer Epidemiol Biomarkers Prev* **4**: 721–725

McGuire WL (1986) Prognostic factors in primary breast cancer. *Cancer Surv* **5**: 527–536

McTiernan A, Thomas DB, Johnson LK and Roseman D (1986) Risk factors for estrogen receptor-rich and estrogen receptor-poor breast cancers. *JNCI* **77**: 849–854

Mendelson JH, Mello NK and Ellingboe J (1981) Acute alcohol intake and pituitary gonadal hormones in normal human females. *J Pharmacol Exp Ther* **218**: 23–26

Mendelson JH, Mello NK, Cristofaro P, Ellingboe J, Skupny A, Palmieri SL, Benedikt R and Schiff I (1987) Alcohol effects on naloxone-stimulated luteinizing hormone, prolactin and estradiol in women. *J Stud Alcohol* **48**: 287–294

Mendelson JH, Lukas SE, Mello NK, Amass L, Ellingboe J and Skupny A (1988) Acute alcohol effects on plasma estradiol levels in women. *Psychopharmacology* **94**: 464–467

*© Cancer Research Campaign 1999*

Mendelson JH, Mello NK, Teoh SK and Ellingboe J (1989) Alcohol effects on luteinizing hormone releasing hormone-stimulated anterior pituitary and gonadal hormones in women. *J Pharmacol Exp Ther* **250**: 902–909
Mobbs BG, Fish EB, Pritchard KI, Oldfield F and Hanna WH (1987) Estrogen and progesterone receptor content of primary and secondary breast carcinoma: influence of time and treatment. *Eur J Cancer Clin Oncol* **23**: 819–826
Nasca PC, Liu S, Baptista MS, Kwan CS, Jacobson H and Metzger BB (1994) Alcohol consumption and breast cancer: estrogen receptor status and histology. *Am J Epidemiol* **140**: 980–987
Potter JD, Cerhan JR, Sellers TA, McGovern PG, Drinkard C, Kushi LR and Folsom AR (1995) Progesterone and estrogen receptors and mammary neoplasia in the Iowa's Women's Health Study: how many kinds of breast cancer are there? *Cancer Epidemiol Biomarkers Prev* **4**: 319–326
Raabe NK, Hagen S, Haug E and Fossaa SD (1998) Hormone receptor measurements and survival in 1335 consecutive patients with primary invasive breast carcinoma. *Int J Oncol* **12**: 1091–1096
Reichman ME, Judd JT, Longcope C, Schatzkin A, Clevidence BA, Nair PP, Campbell WS and Taylor PR (1993) Effects of alcohol consumption on plasma and urinary hormone concentrations in pre-menopausal women. *J Natl Cancer Inst* **85**: 722–727
Romain S, Lainé Bidron C, Martin PM and Magdelenat H on behalf of the EORTC Receptor Study Group (1995) Steroid receptor distribution in 47 892 breast cancers. A collaborative study of 7 European laboratories. *Eur J Cancer* **31A**: 411–417
Romain S, Spyratos F, Goussard J, Formento J-L and Magdelénat H on behalf of the French Study Group on Tissue and Molecular Biopathology (1996) Improvement of quality control for steroid receptor measurements: analysis of distributions in more than 40 000 primary breast cancers. *Breast Cancer Res Treat* **41**: 131–139
Ruder AM, Lubin F, Wax Y, Geier A, Alfundary E and Chetrit A (1989) Estrogen and progesterone receptors in breast cancer patients. Epidemiologic characteristics and survival differences. *Cancer* **64**: 196–202
Singletary KW (1996) Alcohol and breast cancer: interactions between alcohol and other risk factors. *Alcohol Clin Exp Res* **8**: 57A–61A
Stanford JL, Szklo M and Brinton LA (1986) Estrogen receptors and breast cancer. *Epidemiol Rev* **8**: 42–59
Tani E, Nordenskjold B, Humla S and Skoog L (1988) Estrogen receptor content in primary breast carcinomas and their metastases. *Anticancer Res* **8**: 169–172
Teoh SK, Mendelson JH, Mello NK and Skaupny A (1988) Alcohol effects on naltrexone-induced stimulation of pituitary, adrenal and gonadal hormones during the early follicular phase of the menstrual cycle. *J Clin Endocrinol Metab* **66**: 1181–1186
Thorpe SM (1988) Estrogen and progesterone receptor determinations in breast cancer. *Acta Oncol* **27**: 1–19
Toniolo PG, Levitz M, Zeleniuch-Jacquotte A, Banerjee S, Koenig KL, Shore RE, Strax P and Pasternack BS (1995) A prospective study of endogenous estrogens and breast cancer in postmenopausal women. *J Natl Cancer Inst* **87**: 190–197
United States Department of Agriculture (1986) Composition of foods: beverages; raw, processed, prepared. *Agriculture Handbook* No. 8–14. United States Department of Agriculture, Hyattsville, MD
Yoo K-Y, Tajima K, Miura S, Takeuchi T, Hirose K, Risch H and Dubrow R (1997) Breast cancer risk factors according to combined estrogen and progesterone receptor status: a case-control analysis. *Am J Epidemiol* **146**: 307–314
Zeleniuch-Jacquotte A, Toniolo P, Levitz M, Shore RE, Koenig KL, Banerjee S, Strax P and Pasternack BS (1995) Endogenous estrogens and risk of breast cancer by estrogen receptor status: a prospective study in postmenopausal women. *Cancer Epidemiol Biomarkers Prev* **4**: 857–860

 *© Cancer Research Campaign 1999*
